# Supplementary material for: Weight and body composition outcomes with liraglutide in individuals with well-treated hypothyroidism: A retrospective case-control study
Source: PLoS One. 2025 Sep 11;20(9):e0332091. doi: 10.1371/journal.pone.0332091 (PMC12425291; doi:10.1371/journal.pone.0332091)
Supplement: Attachment — Submitted filename: Response to reviewers.docx [file pone.0332091.s001.docx]

Dear Drs. Afagh Hassanzadeh Rad, Matin Mojaveri Samak and Zahra Abbasi Ranjbar,

Thank you for your thoughtful comments. We believe your comments and suggestions strengthen our manuscript and provide more clinically relevant insights.

Reviewer 1: Dr. Matin Mojaveri Samak

Please clarify whether the dose of liraglutide was consistent across all participants or individually adjusted based on clinical conditions. This detail is essential for an accurate interpretation of the weight loss outcomes.

Thank you for your insightful comment. We agree that clarifying the liraglutide dosing is important for accurately interpreting the weight loss outcomes. We have now added this information to the Methods and Discussion sections of the manuscript.

All patients were prescribed the 3.0 mg dose of liraglutide and more than 90% of participants in both groups were able to reach the maximal dose. While some dose titration based on clinical judgment may have occurred, it was not consistently documented in the medical records. However, any such variation is likely to have occurred at a similar rate across both groups.

While a multivariate regression analysis was conducted, certain lifestyle-related variables—such as dietary adherence, physical activity levels, or treatment compliance—may have a significant impact on the results. If such data are available, please include them; otherwise, their absence should be acknowledged in the limitations section.

We agree that other variables, such as dietary adherence and physical activity levels, may have an impact on the final outcomes. We have added this limitation to the discussion section. It is worth noting that even many phase 3 randomized controlled trials of obesity pharmacotherapy, unfortunately, often do not report adherence to lifestyle recommendations.

“ several limitations must be acknowledged due to the retrospective nature of the study, including the potential for selection bias and confounding. For instance, factors such as dietary adherence and physical activity levels may have influenced the outcomes but could not be reliably assessed because data on these variables were not available.”

Some sentences in the Discussion section would benefit from linguistic editing to enhance clarity and fluency.

Thank you for pointing this out. The discussion was reviewed and edited to ensure clarity and fluency.

The title of Figure 1 should include a brief description of the statistical test used.

A brief description has been added.

“Fig 1. Categorical weight loss in individuals with levothyroxine-treated hypothyroidism compared to individuals with normal thyroid function. Outcomes were similar in all categories. Chi-square test was used to compare groups. A p value of <0.05 was considered as statistically significant.”

Please resolve the discrepancies between the list of figures and tables and their corresponding captions within the manuscript.

Thank you for pointing this out. Reviewed and resolved.

Ensure consistent formatting of p-values throughout the manuscript.

Reviewed and resolved

Reviewer 2:

It is recommended to include the levothyroxine dose in multivariate regression model as well. for example up to 50,up to 100 , up to 150...

Thank you for your suggestion.

We ran the model using levothyroxine dose as a linear variable as well as a categorical variable but we did not find any significance in our data. Since levothyroxine dose and TSH could be correlated, including both could cause multicollinearity in multivariate regression. We selected TSH to be included in the multivariate regression because it reflects the degree of thyroid hormone replacement achieved with levothyroxine. We elaborated on this in the manuscript in methods and results.
